# Conditionally activating optical contrast agent with enhanced sensitivity *via* gold nanoparticle plasmon energy transfer: feasibility study

**DOI:** 10.1186/s12951-014-0056-2

**Published:** 2014-12-07

**Authors:** Kyung Aih Kang, Jianting Wang

**Affiliations:** Department of Chemical Engineering, University of Louisville, Louisville, KY 40292 USA; Center for Devices and Radiological Health, Food and Drug Administration, Silver Spring, Maryland 20993 USA

**Keywords:** Optical contrast agent, Fluorescence enhancement, Fluorescence quenching, Gold nanoparticle, Molecular imaging, NanoPPET

## Abstract

**Background:**

Molecular sensing/imaging utilizing fluorophores has been one of the most frequently used techniques in biomedical research. As for any molecular imaging techniques, fluorescence mediated sensing always seeks for greater specificity and sensitivity. Since fluorophores emit fluorescence while their electron energy state changes, manipulating the local electromagnetic field around the fluorophores may be a way to enhance the specificity and sensitivity. Gold nanoparticles (GNPs) are known to form a very strong electromagnetic field on their surface [i.e., surface plasmon field (SPF)], upon receiving photonic energy. The level of fluorescence change by GNP-SPF may range from complete quenching to extensive enhancement, depending upon the SPF strength, excitation and emission wavelengths, and quantum yield of the fluorophore.

**Method:**

Here, we report a novel design that utilizes BOTH fluorescence quenching and enhancement abilities of the GNP in one single nano-entity, providing high specificity and sensitivity. The construct utilizes a specially designed molecular dual-spacer that places the fluorphore at the location with an appropriate GNP-SFP strength before and after exposed to the biomarker. A model system to test the concept was an optical signal mediator activated by urokinase-type plasminogen activator (uPA; breast cancer secreting enzyme).

**Results:**

The resulting contrast agent shows less than 10% of the natural fluorescence but, in the presence of uPA, its fluorescence emission is triggered and emits its fluorescence approximately twice of the natural form.

**Conclusion:**

This study demonstrated that our novel design of an optical contrast agent can be conditionally activated with enhanced sensitivity, using both quenching and enhancement phenomena of fluorophores in the electromagnetic field of the appropriate strengths (in this case, locally generated by the GNP-SPF). This entity is similar to molecular beacon in terms of specificity but with greater sensitivity. In addition, it is not restricted to only DNA or RNA sensing but for any designs that cause the change in the distance between the fluorophore and GNP, upon the time of encountering biomarker of interest.

## Introduction

Molecular imaging using fluorophore as a signal mediator is sensitive, cost-effective, rapid and user-friendly [1-4]. The emission of fluorescence is by the change in electron energy state of the fluorophore, upon receiving photonic energy. Therefore, placing a localized electro-magnetic field near a fluorophore can affect the excitation state of the fluorophore, which may be beneficially used for fluorescence manipulation. A practical means to create this localized field is a surface plasmon field (SPF) generated by a metal nanoparticle [5,6]. Out of metal nanoparticles that can generate strong SPF, gold nanoparticles (GNPs) are most popular, particularly for biomedical research, because of their chemical inertness and well defined surface modification method [7,8].

The level of fluorescence output affected by the SPF depends on the field strength where the fluorophore is placed [9-17]. The two main fluorophore properties that get affected by the SPF are the fluorophore excitation decay rate and quantum yield, and the combination of these two ultimately determines the fluorescence level. The GNP and fluorophore properties that affect the fluorescence output are several, including the wavelengths of the applied (excitation) and emission lights, intrinsic quantum yield of the fluorophore, GNP size, and the distance between the fluorophore and GNP [18-21]. Once the fluorophore for the application is selected, however, the GNP size and the distance between the fluorophore and GNP may be the only two variables to be utilized for the purpose. Theoretical analyses on the resulting fluorescence level affected by the GNP-SPF with respect to the GNP size and the distance between the fluorophore and GNP have been performed by several research teams [22,23]. A detailed analysis on the fluorescence outputs affected by the GNP-SPF for the fluorophores frequently used in biomedical studies has been presented in our previous publications [18,19,24].

**Figure 1:**
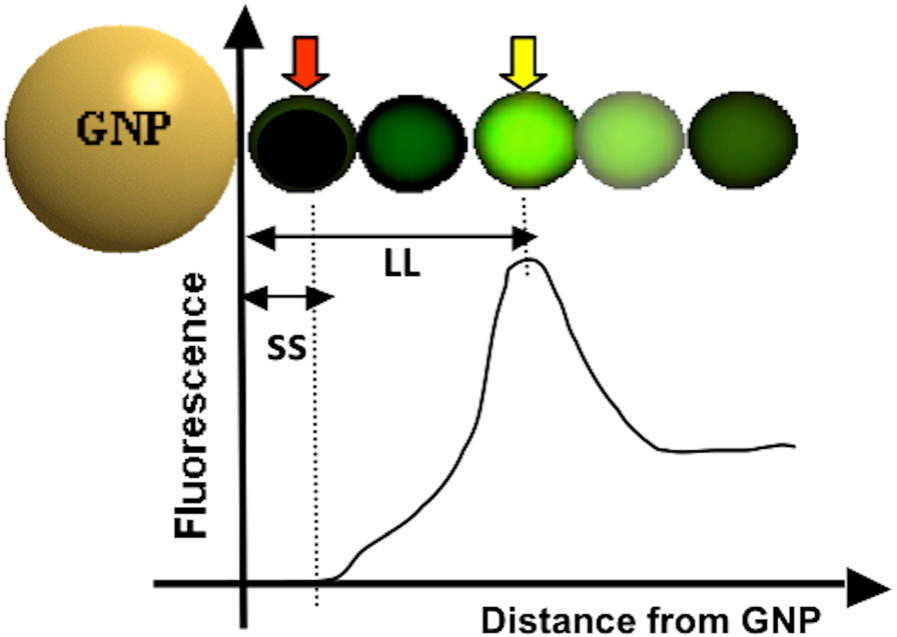
**A schematic diagram illustrating the fluorescence emission level with change in the distance between a fluorophore and a GNP.** Outside the SPF field of a GNP, fluorescence level does not get affected. As the fluorophore gets closer to the GNP, fluorescence is enhanced until it reaches a particular distance (LL). If the distance becomes even closer the fluorescence gets quenched, and on the GNP surface (SS) the fluorescence becomes completely quenched.

Figure 1 is a simple, qualitative illustration of fluorescence intensity of a fluorophore with changes in the distance from a GNP. When the fluorophore is far from a GNP and therefore outside the GNP-SPF, its fluorescence output does not change. As the fluorophore moves inside the GNP-SPF, its fluorescence becomes stronger until it reaches a particular distance (i.e., SPF strength) from the GNP. At this distance, the fluorescence is maximized. After then, as it gets even closer to the GNP, its emission level becomes reduced, and it is completely quenched on the GNP surface. Recently, there have been many publications on utilizing the GNP-SPF for developing more efficacious fluorescence signal mediators for biosensing [25-28] and bioimaging [18,19,24]. They clearly demonstrate that appropriately quenching and enhancing fluorescence emission by GNP-SPF can increase the selectivity and/or sensitivity of molecular sensing, respectively.

Our design, demonstrated here, is to realize a novel fluorophore/GNP complex *via* taking advantage of BOTH fluorescence quenching and enhancing ability of GNP-SPF. The resulting complex is to emit the fluorescence only when it encounters the biomarker of choice at an enhanced level, thus achieving BOTH improved selectivity and greater sensitivity. We named this novel construct NanoPPET (NanoParticle Plasmonic Energy Transfer).

**Figure 2:**
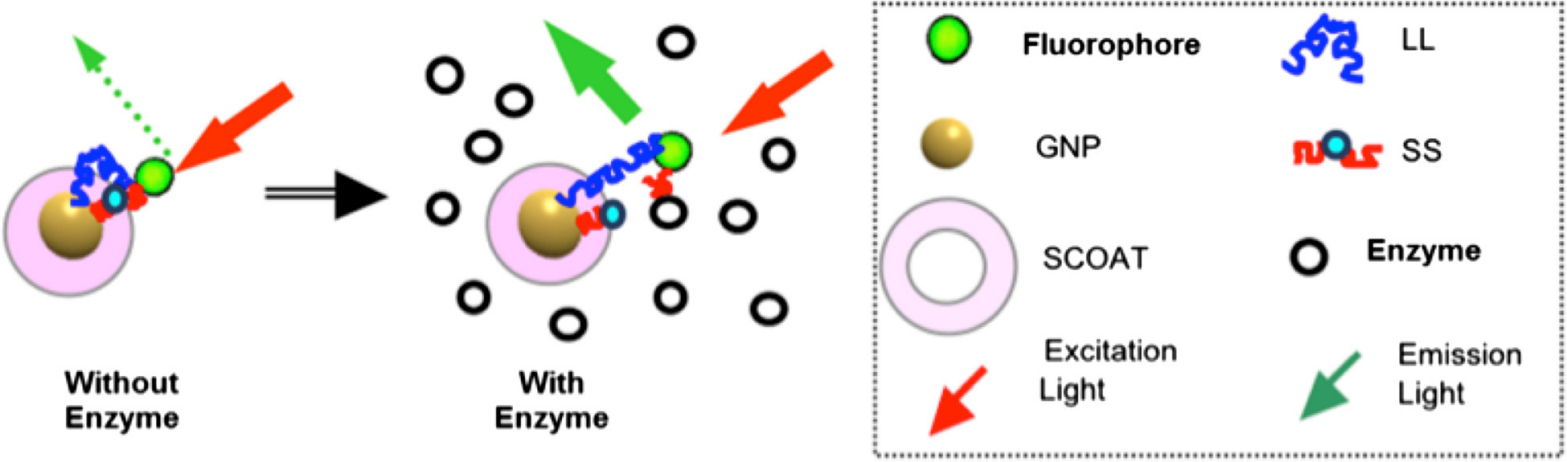
**Enzyme-biomarker triggered, highly sensitive fluorophore/GNP complex.** The complex normally emits little fluorescence because the SS places the fluorophore close to the GNP for fluorescence quenching. When the complex is placed in an environment with the enzyme biomarker, SS is cleaved by the enzyme and the distance between the fluorophore and GNP becomes to the length of LL, resulting in the fluorescence emission at an enhanced level. In this illustration, to simplify the concept, only a set of SS and LL for the GNP are shown. In reality, multiple SS/LL-fluorophore sets are to bind to a single GNP.

Our first model system present here is for sensing a biomarker with enzymatic property. Figure 2 depicts our NanoPPET, enzyme-triggered highly sensitive fluorophore/GNP complex. Briefly, the fluorophore and GNP are connected *via* two spacers, one short and one long. The distance of the short spacer (SS) is designed to be short enough to be able to place the fluorophore within a GNP-SPF sufficiently strong to extensively quench the fluorescence (i.e., distance from the GNP; red arrow in Figure 1). The SS must contain a substrate motif for the enzyme biomarker of interest, so that it gets cleaved when the complex encounters the biomarker. The long spacer (LL) should be a bio-stable and biocompatible sequence at a length that can maximize the fluorescence (yellow arrow in Figure 1).

The fluorescence of the complex is normally minimal because the SS places the fluorophore in close proximity to the GNP, resulting in fluorescence quenching. Once the complex is placed in an environment with the biomarker, the SS is cleaved and, the distance between the fluorophore and the GNP is now determined by the length of the LL, resulting in an enhanced fluorescence level. In this figure, only a single pair of SS/LL is illustrated on a single GNP, to demonstrate the concept. In actual systems, it is suggested to place as many sets as possible, as long as the number of the fluorophore molecules on the GNP is below the level of the intramolecular-fluorescence self-quenching [29] and also the level of the particle aggregation caused by the fluorophores with hydrophobic nature.

Below are detailed descriptions on each component of our model NanoPPET-construct.

1. Our fluorophore was Cypate. It is a near infrared (NIR) fluorophore with the excitation and emission peak wavelengths at 780 and 830 nm, respectively [30]. We selected an NIR fluorophore because NIR penetrates deeper into tissue, which is very important for non-invasive optical imaging. Cypate is an analog of the FDA approved fluorophore, Indocyanine Green (ICG) [4,31]. It is to be non-toxic to animals up to 10 μmol/kg [30], and considered to be biocompatible although it has not yet been submitted for FDA approval. Unlike ICG, Cypate has two COOH- side chains, providing the ability to easily react to other biomolecules. For our study, the COOH- side chains were further modified to –CHO (modified Cypate; mCy) to react to –ONH_2_ group of spacers (Figure 3).

**Figure 3:**
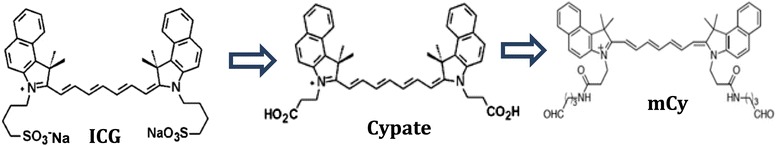
**Structures of ICG, Cypate, and modified Cypate (mCy).**
2. The biomarker selected was urokinase type plasminogen activator (uPA), which is over-expressed by several malignant breast cancer types [32].
3. The GNP surface excluding the Cypate binding sites was covered with self-assembled monolayer of a short, amphiphilic molecule, HS-(CH_2_)_11_-(EG)_3_-OH, where EG is ethylene glycol, and we will call this as surface coat (SCOAT), henceforward. Its hydrophobic part –(CH_2_)_11_- goes to the GNP surface to form tight packing and the hydrophilic part goes to the outside, accommodating better solubility in biofluids. For the NanoPPET, this method of surface packing works better than covering the surface with biopolymers of large molecular weight [33,34], because our complex requires precisely controlled distance between the Cypate and the GNP for the fluorescence manipulation. SCOAT would have minimal interference with the spacers because the spacers are longer and more flexible than SCOAT.
4. The short spacer is to be a peptide sequence containing the substrate motif of uPA.
5. The long spacer is to be a bio-polymer sequence at a length that provides the maximum fluorescence enhancement of Cypate for the selected GNP.

More details on (4) and (5) will follow in the upcoming sections.

Before we designed our ultimate Cypate/spacers/GNP NanoPPET, we needed to validate the integrity of two critical components of the complex: (1) proper fluorescence quenching by using a SS in the Cypate/GNP complex and uPA-triggered fluorescence restoration; and (2) identifying the LL (of a proper length) that can maximize the Cypate fluorescence enhancement. The detailed results for the Cypate/SS/GNP complex and the Cypate/LL/GNP complex were already reported in two of our publications (reference number 19 and 18, for the former and the latter, respectively). Here, a brief summary of the results is provided.

## Previous results

**Figure 4:**
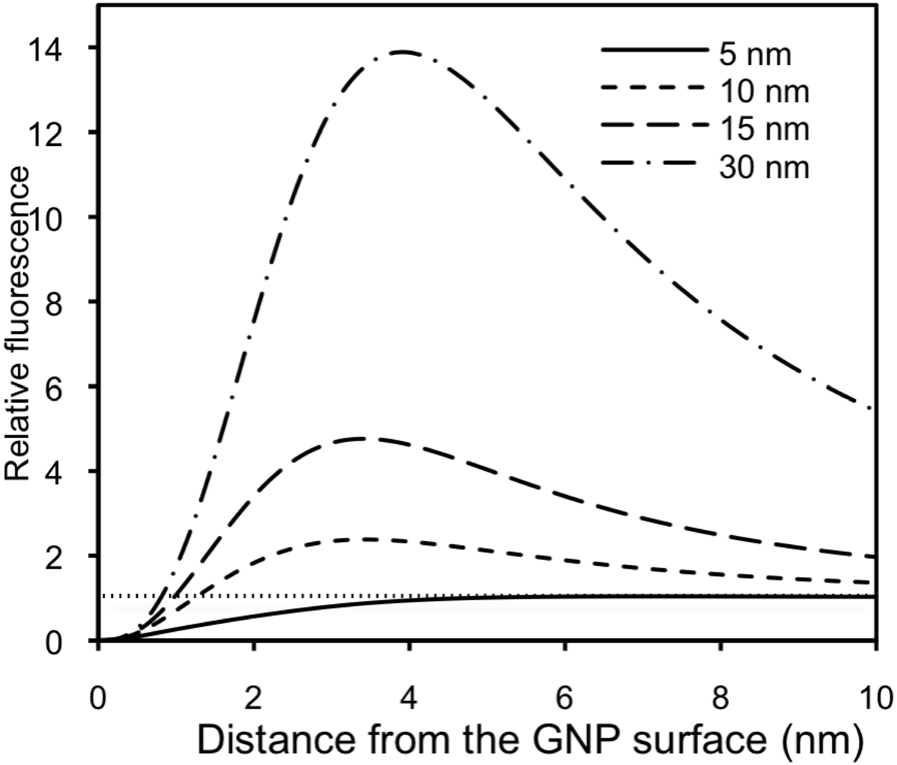
**Theoretically estimated fluorescence level of Cypate (Ex/Em, 780/830 nm) relative the fluorescence without the influence of GNP-SPF, with change in the distance from a GNP at sizes 5, 10, 15, and 30 nm.**

As the first step in developing our NanoPPET, it was necessary for us to determine the optimal lengths for the short and long spacers. They were first theoretically estimated for the Cypate fluorescence level (relative) with respect to the distance from the GNP at various sizes (Figure 4). As can be seen in the figure, for any individual GNP size, there is a distance range for fluorescence quenching (less than 1) and another range for enhancement. Our goal was, therefore, to select appropriate GNP size and two spacer lengths for appropriate quenching and enhancement for the selected GNP size.

### A. Confirming SS integrity for conditionally signaling optical contrast agent

A functional GNP-SS-Cypate complex should possess a SS that (a) contains the uPA substrate motif; (b) is sufficiently short for Cypate fluorescence quenching; and (c) can be properly cleaved upon being exposed to uPA so that the complex restores its fluorescence. The peptide substrate motif for uPA is Gly-Gly-Arg (GGR) sequence [35]. As shown in Figure 4, the SS would perform its quenching role the best when it is as short as possible and, at the same time, it should possess all three functions specified above. After thorough theoretical and experimental investigations, the final SS structure was determined to be SH-(CH_2_)_2_-Gly-Gly-Arg-Gly-Gly-Gly-NH_2_. This sequence included a short hydrocarbon ending with a thiol group for conjugating SS to the GNP, a GGR sequence, and a few extra peptides for maintaining its functional integrity as the substrate after its conjugation to GNP and to Cypate.

**Figure 5:**
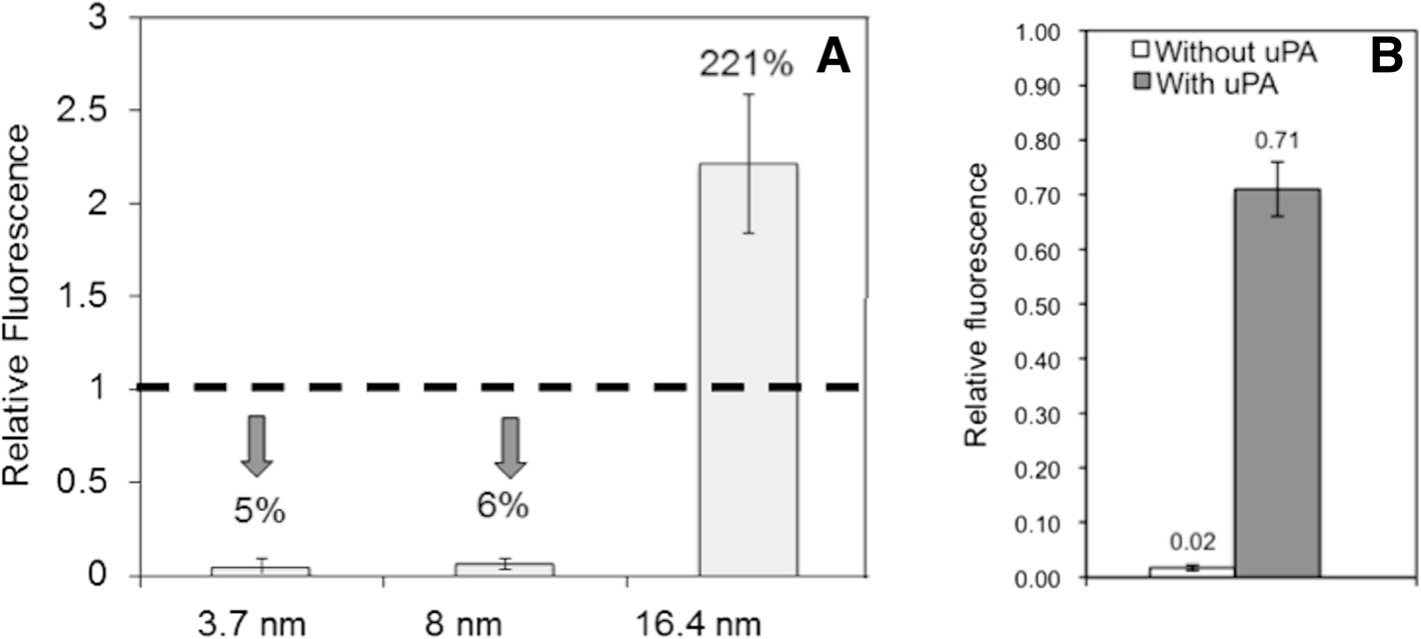
**Confirmation of SS integrity. (A)** Relative fluorescence levels of the GNP-SS-Cypate for the GNP size of 3.7, 8.0, and 16.4 nm. With 3.7 and 8 nm GNPs, the fluorescence is quenched significantly. For 16.4 nm, the fluorescence is enhanced, instead. **(B)** Fluorescence of 8.0 nm GNP-SS-Cypate before and 5 minutes after adding uPA. Fluorescence is restored as uPA cleaves SS.

We then tested the SS for GNP sizes at 3.7, 8, and 16.4 nm, and 3.7 and 8 nm GNPs showed satisfactory performances for quenching (Figure 5A). For the 16.4 nm GNP the fluorescence was enhanced instead, and therefore this size may not be used for our purpose. We selected 8.0 nm for our further studies since we later would have to use the same size GNP for the fluorescence enhancement and the enhancement level is greater for larger GNPs. Figure 5B shows the normalized fluorescence levels before and after the 8.0 nm GNP complex was exposed to uPA for 5 minutes. It demonstrates the feasibility of our design for the uPA-triggered fluorescence emission.

### B. Confirming LL functional integrity for Cypate/GNP complex with enhanced sensitivity

**Figure 6:**
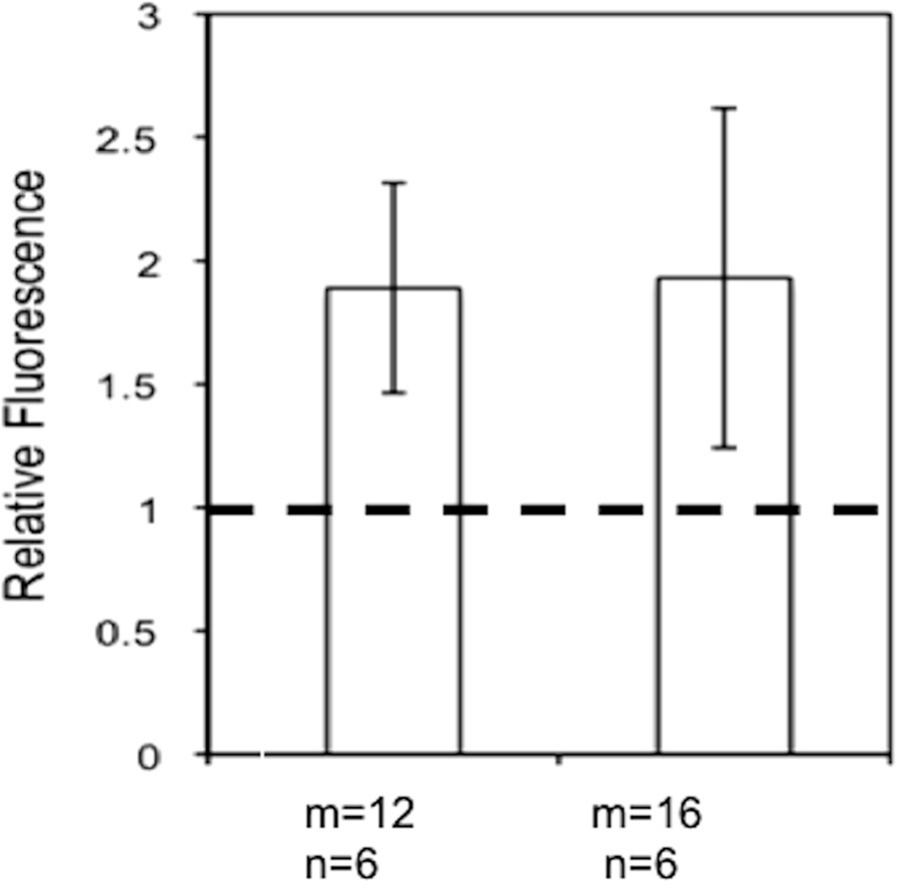
**Relative fluorescence of GNP-LL-Cy conjugated to 8 nm GNP, for the LL spacer that provids good flourescence enhancement for our purpose.** Fluorescence is enhanced approximately twice of the level by Cypate alone.

For the second component of our NanoPPET, a proper LL for Cypate/GNP (8 nm) complex emitting maximal fluorescence needed to be identified. We designed the LLs with amphiphilic molecular chains, similar to the materials used for SCOAT, so that we could mix them at an appropriate ratio and conjugated them together on the GNP surface. They were HS-(CH_2_)_m_-(EG)_n_-ONH_2_ and by changing the number *m* and *n*, the length of the spacer was varied. For easier reaction with –ONH_2_ groups, mCy with –CHO groups was used instead of Cypate with –COOH groups (Figure 3). Our results show that the chains with m = 12 or 16 and n = 6 offered the maximum fluorescence enhancement, which was 200% of the fluorescence level by mCy alone (Figure 6) and also similar to the simulation result shown in Figure 4. We selected m = 12 for further studies, as its structure is closer to our SCOAT and should mix well with SCOAT, as a self-assembled monolayer.

## Results and discussion

The NanoPPET, shown in Figure 2, is to integrate the functions of both complexes with short spacer and long spacer, i.e., it normally does not fluoresce but, in the presence of uPA, it is to emit fluorescence at an enhanced level. All experiments were performed at least three times and the results were presented with the mean and the standard deviation for their validity.

### A. Initial attempt for two spacer-NanoPPET

**Figure 7:**
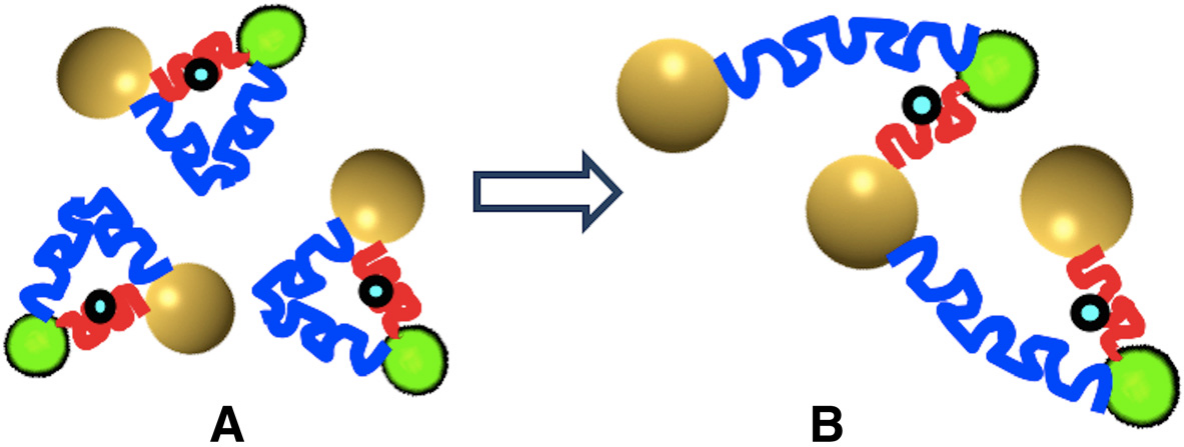
**An issue in designing two-spacer NanoPPET. (A)** Idealistic two spacer conjugation for Cypate-dual spacer–GNP complex and **(B)** the product likely formed during the Cypate and GNP reacting with two spacers: GNP cross-linking and precipitation.

Our first approach for realizing the NanoPPET was that Cypate and the GNP (8 nm) would be connected *via* the two spacers, an *SS* and an *LL*. The two spacers were, therefore, designed to be linked using the two –COOH groups of Cypate. Once a Cypate molecule was conjugated to each end of *SS* and *LL* then the other ends of the spacers were to be bound to the GNP surface *via* their thiol groups (Figure 7A).

In the process of realizing this design we encountered a few major problems. First, in this design, Cypate needs to be conjugated to two different spacers. It was, however, difficult to ensure two –COOHs of a Cypate molecule reacted with one SS and the other with LL. Secondly, assuming that the LL-Cypate-SS were properly formed, this complex needed to be conjugated to the surface of a single GNP and this task was found to be almost impossible: Endings of both SS and LL had reactive thiol groups and the two spacers were different in length. Therefore, the reaction could produce a combination of several undesired products. For examples, only one spacer would be attached to a GNP and the other spacer is free; the spacers would be attached to two different GNPs; and multiple GNPs can be connected with multiple LL-Cypate-SS, resulting in cross-linking. Slight cross-linking may be tolerable and the fluorescence may still be quenched as long as Cypate was conjugated to GNP *via* the *SS*. Severe cross-linking may result in product aggregation (Figure 7B) and precipitation. In fact, due to the problems mentioned above, we experienced serious particle aggregation and precipitation even for the reaction with a very low reactant concentration.

### B. NanoPPET with new dual spacer design

To resolve the problems addressed above, a completely new approach was implemented with a goal that a single Cypate molecule should be reacted with an SS and an LL, and that the SS/LL pair should be conjugated on a single GNP. Our new approach was, therefore, reducing the total reaction sites between the SS/LL pair, and Cypate and GNP, from the original four sites to two, while one new site is conjugated to Cypate and the other, to a GNP. In addition, the two reaction mechanisms must be different, avoiding cross-linking of resulting particles.

**Figure 8:**
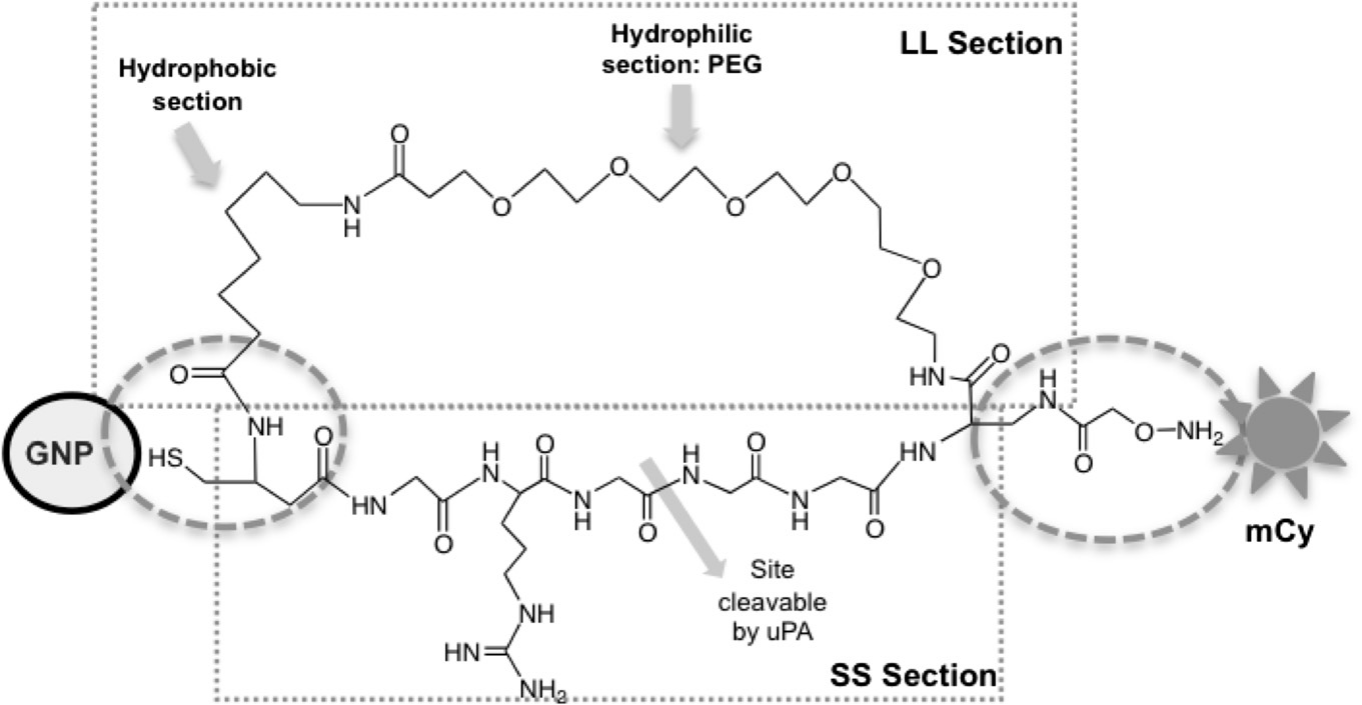
**Chemical structure of the new dual spacer.** The modification was reducing the number of binding sites from four sites (two between the spacers and Cypate, and two between spacers and GNP) to two (one to Cypate and one to GNP), by converting the design to a ring-shaped dual spacer (rSP).

The main feature in the new design was creating a ring-shaped dual spacer (rSP), which still possess the properties of both SS and LL, but with only two reaction sites, one site to a GNP specifically and the other to Cypate (Figure 8). To facilitate the synthesis of an appropriate rSP, the SS and LL sections were slightly modified. Our original SS was HS-(CH_2_)_2_-GGRGGG-NH_2_. At the –SH end, HS-(CH_2_)_2_-NH- was replaced by HS-CH_2_-CH(NH_2_)-C(O)-, which provides a side group for connecting the new LL section of rSP.

The original ending –NH_2_ was replaced by –NH-CH(COOH)-CH_2_-NH-C(O)-CH_2_-O-NH_2_, which has a side group –COOH for connecting the other end of LL, and the –O-NH_2_ ending enables an easy reaction to mCy (modified Cypate with –CHO group). However, the change introduced an extra length of [−CH(COOH)-CH_2_-NH-C(O)-] and, to keep the length of the new *SS* similar to the original one, while maintaining its function as a uPA substrate, one Glycine was removed from the original SS, i.e., −GGRGGG- became –GRGGG-.

The original *LL* was HS-(CH_2_)_12_-(OCH_2_CH_2_)_6_-ONH_2_. In the new design, Cysteine [HS-CH_2_-CH(COOH)-NH_2_] providing an –SH group added an extra length of –CH_2_-CH(COOH)-NH-. For the –ONH_2_ end, using H_2_N-CH(COOH)-CH_2_-NH-C(O)-CH_2_-O-NH_2_ results in the extra length of C(O)-CH(NH)-CH_2_-NH-C(O)-CH_2_. The hydrocarbon chain and PEG are connected through amide-bond, which also introduces extra length [−NH-C(O)-]. To compensate these length increases, the –CH_2_- number was reduced from 12 to 6, and EG number was reduced from 6 to 5.

The resulting rSP became cyclo [DAP(Aoa)-PEG5-(8-aminooctanoicacid)-Cys-Gly-Arg-Gly-Gly-Gly] (Figure 8). The *r*SP has one –SH group at the one end for the GNP surface, and –ONH_2_ group at the other end for reacting with mCy. For the GNP size, 8 nm GNPs were selected, as determined in the previous studies with the Cypate/SS/GNP and Cypate/LL/GNP complexes. rSP and SCOAT were mixed at 1:9 molar ratio and reacted with 8 nm GNPs. Once the rSP-GNP complex was formed and purified by dialysis and centrifugation, the GNP in the resulting complex solution was quantified by the light absorption at 520 nm. The purified rSP-GNP was then reacted with mCy and the resultant was purified by dialysis and centrifugation. The GNP and mCy in the final product were quantified by the light absorption at 520 nm and 780 nm, respectively.

**Figure 9:**
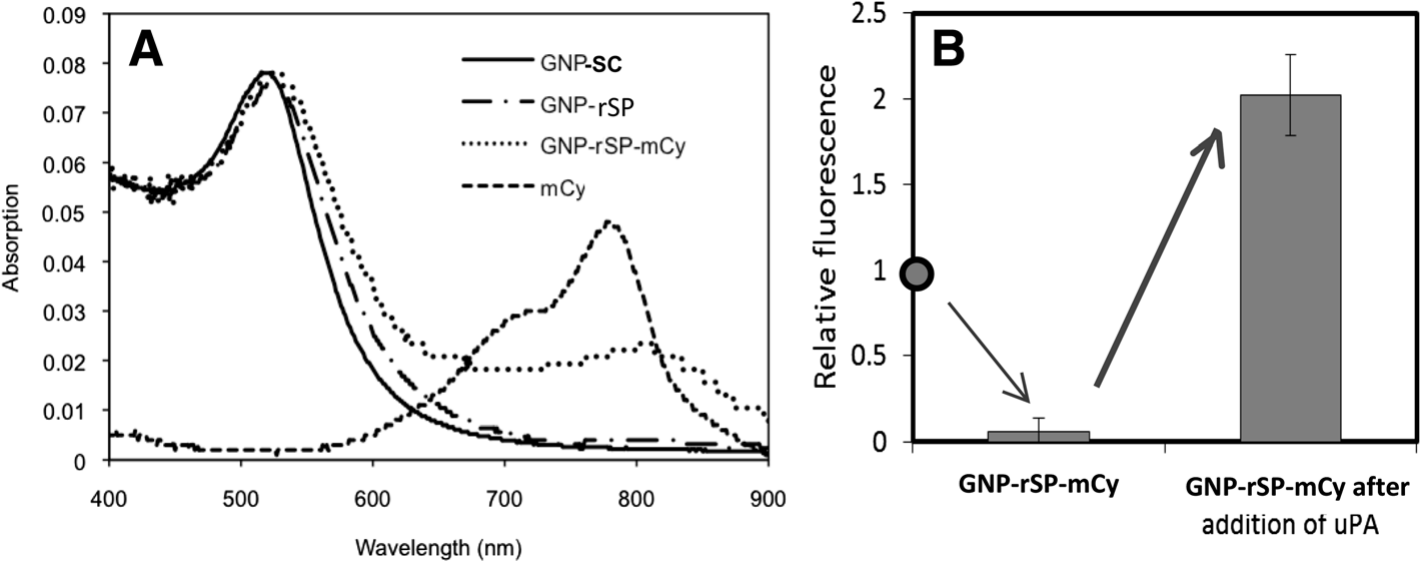
**Characterization of resulting uPA-specific NanoPPET. (A)** Absorption spectra of 8.0 nm GNP, GNP-SCOAT, GNP-rSP-mCy, and mCy and **(B)** Fluorescence emission levels for free mCy, GNP-rSP-mCy complex, and uPA treated complex.

Figure 9A shows the absorption spectra of GNP, mCy, and GNP-*r*SP-mCy. The GNP-*r*SP-mCy spectrum shows both GNP and mCy absorption peaks, confirming the conjugation of mCy to GNP *via* rSP. The ratio between mCy to GNP ratio for the final product GNP-*r*SP-mCy was found to be approximately 110:1.

The purified GNP-*r*SP-mCy was then reacted with uPA and its fluorescence was measured and compared with that without uPA. Figure 9B shows the fluorescence level of the GNP-rSP-mCy before and after reacting with uPA, relative to the mCy fluorescence at the same concentration without GNP. The fluorescence of GNP-*r*SP-mCy before the reaction with uPA was less than 7% of mCy alone, very close to the one for GNP-SS-Cypate shown in Figure 5. Upon applying uPA the fluorescence of reacted GNP-*r*SP-mCy started to increase, and after 5 minutes it became up to 2.0 times of mCy alone, which is the enhancement achieved by the 8.0 nm GNP–LL-Cypate (Figure 6) and also very close to the simulation results (Figure 4). The results confirmed the feasibility of NanoPPET concept, which utilizes both fluorescence quenching and enhancing properties of the GNP-SPF in one single entity to enhance both specificity and sensitivity. The NanoPPET is similar to a molecular beacon in terms of its specificity but not restricted only to the nucleotide sensing, and it can also provide an enhanced sensitivity [36], although further studies should be done to compare the performances of the two.

## Conclusion

We have demonstrated a method for developing a novel optical contrast agent, NanoPPET, by appropriately utilizing GNP-SPF, for biomarker-specific fluorescence emission at an enhanced level. A model system of uPA-triggered Cypate fluorescence emission at an enhanced level was experimentally confirmed. Our NanoPPET model exhibited less than 10% fluorescence before it is exposed to uPA and once exposed, the fluorescence level became 200% of the original level. Although this particular design is for the biomarker uPA, the NanoPPET concept can be used for any biomarker that causes a change in the distance between a fluorophore and a GNP. The properties of NanoPPET is similar to a molecular beacon, featuring high specificity, but with further benefits of not being restricted to the DNA or RNA sensing and, more importantly, providing an enhanced signal intensity. GNPs can also provide the platform for including other molecular entities, such as additional targeting molecules or therapeutic agents, as well as being able to be used as X-ray/CT contrast agent with no further modification.

The next step for our studies will be testing the NanoPPET for uPA secreting celllines and animal models.

## Materials and methods

### A. Materials and instruments

Gold nanoparticles (GNPs; 8 nm) coated with citric acid in aqueous solution (Ted Pella; Redding, CA) was reacted with (1-Mercapto-11-undecyl) tri(ethylene glycol) [HS-(CH_2_)_11_-(CH_2_CH_2_O)_3_; MW, 380.58] (ProChimia Surfaces; Poland) for coating the GNP surface. Urokinase-type plasminogen activator (uPA) was purchased from Innovative Research (Novi, MI). The ring-shaped spacer (rSP) was provided by Abgent (San Diego, CA) through customized synthesis.

Dialysis of various GNP complexes was done with Slide-A-Lyzer Dialysis Cassettes (20 K MW Cut-Off, Thermo Scientific; Rockford, IL). Centrifugation was done using Eppendorf 5415 R Centrifuge (Eppendorf AG; Hamburg, Germany). Absorption spectra of GNPs were obtained using Beckman DU520 spectrometer (Beckman Instruments, Inc.; Fullerton, CA). A sonicator (Sonic Dismembrator; Fisher Scientific; Chicago, IL) was used to disperse GNPs in solutions after centrifugation.

GNP size was analyzed by a dynamic light scattering (DLS) particle size analyzer (90Plus/BI-MAS; Brookhaven Instruments Co.; Holtsville, NY). Fluorescence was measured by Spectra Gemini XPS fluorometer (Molecular Devices Corp.; Sunnyvale, CA) in a 96-well Uniplate (Whatman; Florham Park, NJ) at the excitation and emission wavelengths of 780 and 830 nm, respectively.

### B. Methods

GNP-rSP-mCy was produced by the procedure described below:

1. Colloidal GNP solution was concentrated ~10 times by centrifugation at 13,000 RPM for 60 mins followed by re-dispersion in DI water; The GNP was quantified by the light absorption at 520 nm;
2. rSP and SCOAT (SC) were mixed at 1:9 ratio and diluted in ethanol at the same volume of the GNP solution to be added;
3. GNP solution was added to the rSC/SC mixture solution drop-wise with stirring; the mixture was stirred for 4 hrs at room temperature. According to Duchesne et al. [33], a tightly packed, mixed monolayer of SC and peptide on GNP surface corresponds to 3.6 molecule/nm^2^-GNP surface area. Here, for the amount of *r*SP and SC, 60 molecule/nm^2^-GNP surface-area (excess by ~15 times) was used in the reaction;
4. After the reaction, the solution was placed in a dialysis cassette (20 K MW cut-off) and the cassette was placed in 2 L of DI water in dark with stirring, overnight.
5. The dialyzed sample was then centrifuged at 13000 RPM for 60 min. The pellet was re-dispersed in 1 mL of DI water and sonicated for 5 minutes.
6. mCy was dissolved in ethanol at 2.5 mM; a blocking agent, IN-(CH_2_)_2_-ONH_2_ (BLK) was added to mCy solution at a 1:1 molar ratio and reacted in dark, at room temperature, overnight. Addition of BLK is to block one of the two reactive –CHO groups of mCy, and it also increases the hydrophicility of mCy.
7. mCy-BLK was added to the solution from step (5) at 0.3 molecule/nm^2^-GNP surface and reacted with stirring, for 4 hrs, in dark, at room temperature.
8. The solution from step (7) was dialyzed in 2 L of DI water with stirring, in dark, overnight, to remove unreacted mCy;
9. The dialyzed sample was then centrifuged at 13000 RPM for 60 min. The pellet was re-dispersed in 1 mL of DI water and sonicated for 5 minutes.
10. The GNP and mCy in the solution were quantified by UV-Visible spectroscopy.
11. The NanoPPET solution resulted from step (10) was diluted to 50–100 nM mCy concentration, and placed in a 96-well plate. Multiple wells were filled with duplicates of NanoPPET solution and control samples with mCy-BLK at the same mCy concentration, at 200 μL for each well. Small volume (5 μL) of uPA solution was added to the wells to result in a concentration of 1030 units/ml (i.e., a sufficient amount). The fluorescence of the samples was measured before and after adding uPA, until little change in fluorescence was observed. Fluorescence of samples without uPA addition, but with the same volume (5 μL) of water addition was also measured as a negative control.

## Abbreviations

BLK: Blocking agent
CT: Computed tomography
DI: De-ionized
DLS: Dynamic light scattering
FDA: Food and drug administration
GNP: Gold nanoparticles
ICG: Indocyanine green
LL: Long spacer
mCy: Modified cypate
NanoPPET: Nano particle plasmonic energy transfer
rSP: ring-shaped dual spacer
SCOAT/SC: Surface coat
SPF: Surface plasmon field
SS: Short spacer
uPA: urokinase-type plasminogen activator
